# Our lives in boxes: perceived community mediators between housing insecurity and health using a PHOTOVOICE approach

**DOI:** 10.1186/s12939-019-0943-0

**Published:** 2019-03-27

**Authors:** Hugo Vásquez-Vera, Ana Fernández, Ana M. Novoa, Lucía Delgado, Joaquín Barcala, Carlos Macías, Carme Borrell, Photovoice Working Group of Public Health Agency of Barcelona

**Affiliations:** 1 Agència de Salut Pública de Barcelona, Plaça Lesseps 1, ES-08023 Barcelona, Spain; 2 Department of Experimental and Health Sciences, Universitat Pompeu Fabra, Barcelona, Spain; 3 CEES-Departamento de Salud Pública, Universidad de La Frontera, Temuco, Chile; 4 CIBER de Epidemiología y Salud pública (CIBERESP), Madrid, Spain; 5 Institut d’Investigació Biomèdica (IIB-Sant Pau), Barcelona, Spain; 6 Plataforma de Afectados por la Hipoteca de Barcelona, Barcelona, Spain

**Keywords:** Housing insecurity, Foreclosure, Evictions, Photovoice, Mediators, Pathways

## Abstract

**Background:**

While the negative effects of housing insecurity on health are well known, the mechanisms and mediators of these effects have been less well studied. The aim of this study is to identify perceived mediators involved in the relationship between housing insecurity and health.

**Methods:**

We used a participatory action research approach, the Photovoice methodology. It promotes a reflective process where participants critically discuss housing insecurity and human health and make recommendations to find solutions for the issues identified. This study was conducted with 18 members of the Platform for People Affected by Mortgages who were living in a situation of housing insecurity in Barcelona during the first half of 2017.

**Results:**

Participants took 990 photographs, of which 147 were printed for analysis in discussion sessions. 109 of these photographs were then selected for categorization by the participants. 11 major categories emerged, representing various factors related to housing insecurity and health. Most categories were acknowledged as possible mediators of the housing/health problem, including: psychological changes; housing-related material aspects; health-related behaviors; eviction; harassment by financial institutions; and family, neighbors and social network. Others were considered as modifiers that could alter the effects of housing insecurity on health. Co-existing determinants may interact with housing insecurity, thereby increasing negative effects on health.

**Conclusions:**

Through this participatory approach, the Photovoice project gives insight into the mechanisms underlying the relationship between housing insecurity and human health, and provides valuable recommendations to combat this serious public health issue.

**Electronic supplementary material:**

The online version of this article (10.1186/s12939-019-0943-0) contains supplementary material, which is available to authorized users.

## Background

Housing is a key social determinant of health that includes both physical and socioeconomic dimensions [1–4]. This latter dimension is related to several aspects such as housing affordability and secure tenure, which allow people to have a stable home, a place that protects privacy, contributes to physical and mental health and supports the development of a life plan and social integration of its inhabitants [5]. A lack of these features can lead to housing insecurity due to economic barriers, which includes housing-related situations that interfere with the normal development of personal and family life, and are linked to the level of economic and legal security of residential status. It includes the threat of eviction, unsecure housing tenure (e.g. illegal occupation) and doubling-up (i.e. being forced to live with relatives or friends because of a lack of one’s own dwelling) [6–9].

Systematic reviews published in recent years agree that housing unaffordability, foreclosures and the eviction process can have negative consequences for both mental health (depression, anxiety, and suicides) and physical health (poor self-reported health, high blood pressure, child maltreatment) [10–12]. There is also evidence that people affected by these processes are more likely to develop unhealthy behavior, such as smoking and leading a sedentary lifestyle [12].

The Spanish housing market was strongly affected by the ‘subprime’ property crisis in 2007. Due to the increased unemployment, thousands of families were forced to make monthly mortgage and rent payments that they could not afford, leading to a rise in foreclosure, eviction and other types of housing insecurity, such as squatting [13]. Between 2008 and 2017, 725,259 foreclosure proceedings were initiated and 369,152 evictions were executed [14]. This situation severely affects people’s health. For instance, a study conducted in 2014 in Spain reported that the prevalence of poor self-reported health was 55.6% in women and 39.4% in men suffering housing insecurity, and 19.2 and 16.1% in the general population. In addition, the prevalence of poor mental health among people under housing insecurity was 90.6% in women and 84.4% in men, compared to 15.5 and 10.2% in the general population [15]. Feelings of personal failure, shame and stigma are common among people affected by foreclosure and eviction in societies - as the Spanish one - where there is a hyper-commodification of housing, and housing tenure security is considered as an indicator of being a “good citizen” [16, 17].

In response to this crisis, several social organizations have been fighting for housing rights, the most important of which is the Platform for People Affected by Mortgages (PAH). Its objectives are to: stop evictions, obtain reasonable relocation alternatives, and to promote legislative initiatives aimed to increase the social housing stock, ensure basic amenities such as electricity, gas and water, and restore renting to a secure and affordable type of housing tenure [18, 19]. Currently, Barcelona’s PAH has around 120 members, although there is not an accurate register of participation. Aproximately 80 people attend the PAH assemblies every week.

While the negative effects of housing insecurity on health are well known, the mechanisms and mediators underlying these effects have been less well studied. In general, the published evidence focuses on psychosocial pathways and how feelings of personal failure, guilt, fear and insecurity can lead to psychological distress and poor mental health [7, 20–22]. Others have reported that adopting unhealthy behaviors could mediate the link between housing insecurity and several diseases [16, 23, 24]. However, the evidence on this topic remains scarce, and several gaps must be filled to develop a proper conceptual framework on housing insecurity and its effects on health (e.g. neo material mechanisms involved, the role of other related social determinants, and the inequality axes).

The aims of this participatory action research are: i) to identify perceived mediators of the relationship between housing insecurity and health among affected groups with the PAH; ii) to promote a reflective process where participants critically discuss housing insecurity and health based on photographs that they have taken; iii) to make recommendations for tackling the pathways identified, and improve the health of people affected by housing insecurity; and iv) to inform policy makers about these recommendations. This paper identifies perceived mediators of the relationship between housing insecurity and health and summarizes the proposed recommendations.

## Methods

### Design: Photovoice, a participatory action research

This study employs a Participatory Action Research (PAR) approach, which considers community members, researchers and other stakeholders as equals throughout the research and action processes. A community-based knowledge platform is created, with the ultimate aim of improving the lives of the participants [25, 26]. One of the branches of PAR is Photovoice, a tool used in participatory research with “which people can identify, represent and enhance their community through a specific photographic technique” [27]. It encourages participants to take photographs that represent their life experiences and to tell the stories behind these photographs. Through a critical dialogue process based on their personal and collective knowledge, the participants identify the main issues under discussion, namely the mediators that link housing insecurity to health, and propose recommendations to address them [25, 28].

### Study setting and participants

This study was conducted with members from the PAH living in the metropolitan area of Barcelona during the first half of 2017, and who are, or have been, in some of the aforementioned housing insecurity situations.

To represent all agents involved, a steering committee was formed at the beginning of the study and consisted of four researchers from the Public Health Agency of Barcelona, two members from PAH, and the project’s photographer. This group led and organized the entire process.

Recruitment began two weeks prior to the first meeting with the participants. It took place at the PAH headquarters in Barcelona after a brief presentation of the project to the general PAH assembly. Participants were selected using the snowball technique by PAH members of the steering committee, either at the meetings themselves, or via social media. The inclusion criteria were: > 18 years old; living or having lived under the threat of eviction, illegal occupation or doubling-up; able to operate a camera; and willing to attend a two-hour training session and five 90 min discussion sessions.

All participants gave informed written consent and the study was approved by the Ethics Committee of Parc de Salut Mar, Barcelona, as part of the project named *“Aproximación cualitativa a los mediadores entre la seguridad residencial y sus efectos en salud”* (n° 2017/7287/l).

### Photovoice steps

Photovoice activities were conducted between April and July 2017. We set up two groups of 9 participants each: Group 1 consisted of 5 women and 4 men, and Group 2 consisted of 6 women and 3 men. Both groups attended six weekly sessions of approximately two hours at the PAH headquarters in Barcelona. Meeting days and times were decided by each group independently.

A total of 9 sessions were conducted, 6 by each group independently and 3 joint sessions. In each session, 2 facilitators from the steering committee explained the aims of the session, encouraged the involvement of every participant and led the discussions. In addition, 2 non-participant observers took field notes to record nonverbal language and key ideas that emerged, as well as the dynamics and functioning of each session. Sessions 2 to 6 were audio recorded and transcribed with the permission of the participants.

The first session consisted of training, including: a) an overview of the study (objectives, Photovoice process, ethical issues, expected benefits); b) a workshop conducted by a professional photographer to learn how to use the digital cameras given to the participants, as well as basic technical, esthetical and ethical aspects of photography; c) the administration of a brief anonymous survey to gather basic socioeconomic information of the participants; and d) signing of the consent form by each participant. Participants were asked to photograph what they considered to be the mechanisms and pathways that link housing insecurity and health using the following instruction: “photograph the things that have changed in your daily life due to your current residential status, and which are affecting (or might affect) your health or well-being”.

Participants brought their photographs to sessions 2 to 5. They chose three to five pictures, which were printed out and discussed. The main purpose of the second session was to reinforce the ideas to be captured by the participants’ photographs, for the photographer to give feedback to participants concerning technical, esthetical and ethical issues, and to start to discuss the photographs. During sessions 3 to 5 the participants discussed and shared their photographs with the group. The SHOWED method was used to guide the discussion, including five questions: What do you see here? What is really happening? How does this relate to our lives? Why does this problem or strength exist? What can we do about it? [27].

In the sixth session the participants brought their final five selected photographs that best reflected the issues each was trying to capture and had already been discussed in previous sessions. Participants then grouped all the selected photographs to establish categories in order to represent different mediators involved in the relationship between housing insecurity and health.

Finally, in sessions 7 to 9, participants from the two groups worked together to share and select the final photographs and categories, and proposed recommendations to tackle the identified factors.

### Data analysis, and quality of data

Data analysis began with the selection, categorization and discussion of the photographs by the participants during the sessions. When both groups finished their meetings, four members of the steering committee (HV, CB, AN, AF) devised an analysis of the themes and content using the transcripts of the sessions. These four members identified and coded themes using the categories proposed by participants as a frame of reference. The participants then convened to compare and discuss differences in the analysis made by the four members of the steering committee and the two groups of participants. Themes were then re-coded and classified, identifying common patterns and convergences and divergences in data through an iterative process of constant comparison. Finally, the proposed categories were refined and enhanced by the other members of the steering committee and the participants of both groups in a devolution session (session 7).

In order to ensure data quality, the information was verified using the field notes, the transcripts from each session, and related literature.

## Results

**Fig. 1:**
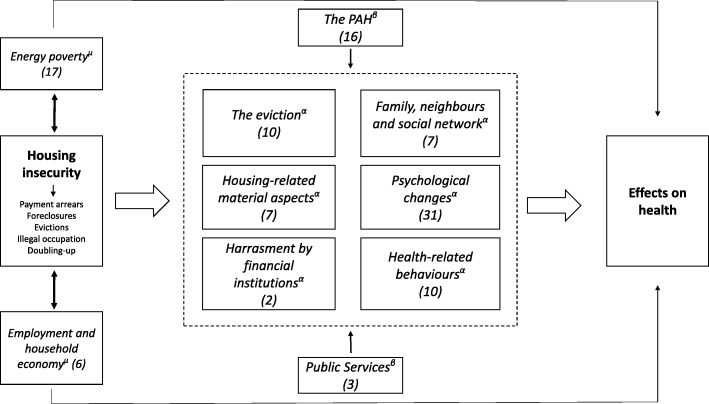
Mediators^α^, modifiers of the mediators^β^ and co-existing determinants^μ^ involved in the relationship between housing insecurity and health (in brackets the number of selected photographs by each category)

The socio-demographic characteristics of the participants are shown in Table 1. A total of 990 photographs were taken, of which 147 were printed for analysis in discussion sessions. Of these photographs, 109 were selected to be categorized by participants. Eleven major categories emerged that represent different factors related to housing insecurity and health (Fig. 1). Most were acknowledged as possible mediators (“psychological changes”; “housing-related material aspects”; “health-related behaviours”; “eviction”; “harassment by financial institutions”; and “family, neighbours and social network”), while others, such as “the PAH” and “the response of public services”, were considered as modifiers that could vary the effects of housing insecurity on health. Two co-existing determinants emerged (“employment and household economy”; and “energy poverty”) which, while not recognized as mediators, might interact with housing insecurity by increasing its negative effects on health. Finally, participants included two categories called “the current housing context” and “stressful life events”, which were described as relevant although they are not directly involved as mediators between housing insecurity and health.

**Table 1:** Socio-demographic characteristics of participants

	Female (*n* = 11)	Male (*n* = 7)	Total (*n* = 18)
Age			
*30 or less years-old*	1	–	1
*31 to 45 years-old*	2	3	5
*46 to 60 years old*	7	3	10
*61 or more years-old*	1	1	2
Country of origin			
*Spain*	4	4	8
*Morocco*	2	1	3
*Peru*	2	–	2
*Honduras*	2	–	2
*Bolivia*	–	1	1
*Ecuador*	–	1	1
*Italy*	1	–	1
Educational level			
*Primary or less*	2	3	5
*Secondary*	7	3	10
*College*	2	1	3
Employment status			
*Employed*	2	3	5
*Unemployed*	7	2	9
*Retiree or pensioner*	2	2	4
Type of housing insecurity			
*Due to mortgage arrears*	6	5	11
*Due to rent arrears*	3	2	5
*Illegal occupation of a dwelling*	2	–	2

### Psychological changes

**Fig. 2:**
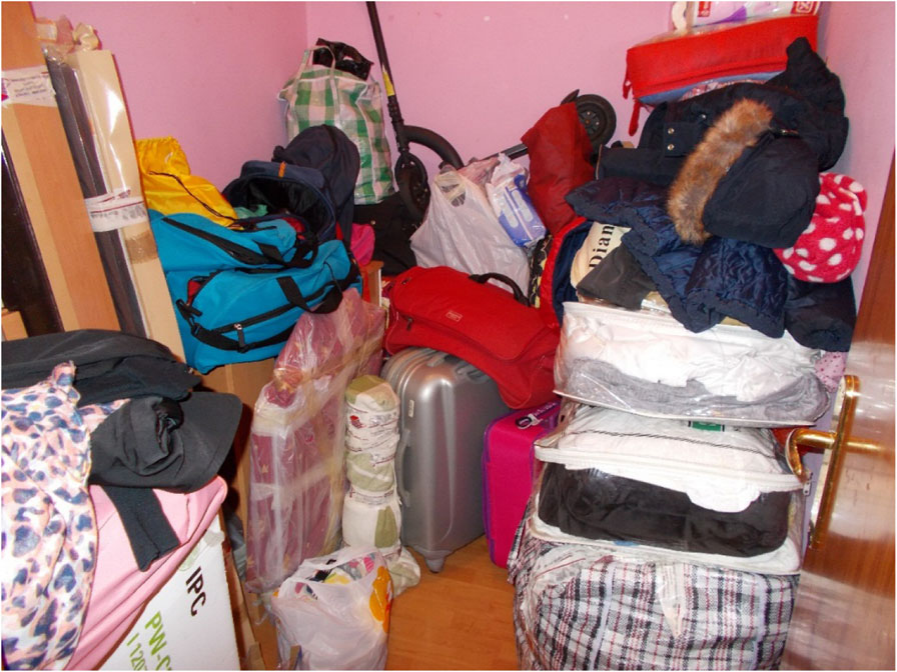
Our lives in boxes (R.N. a 34-year-old women)

Psychological changes and negative feelings related to housing problem were one of the most photographed topics. Participants reported that feelings such as shame, guilt, uncertainty, fear and powerlessness, as well as a lack of control of their daily lives, negatively influenced their wellbeing and health. Uncertainty and fear were the most photographed emotions, which both groups depicted in the photograph, “our lives in boxes” (Fig. 2). This illustrates the threat of being evicted at any time while also not being able to plan one’s life in the dwelling because of housing instability. For instance, C.P., a 50-year-old woman said: “…My apartment is already auctioned! I have the eviction, and I’m afraid that at any moment they will come and take me out onto the street. That’s why I say, look, I will pack my bags, my clothes, the main things. ... and this causes me anxiety, insomnia, nervousness, that any day they will knock on the door and the police will take me out”.

### Housing-related material aspects

**Fig. 3:**
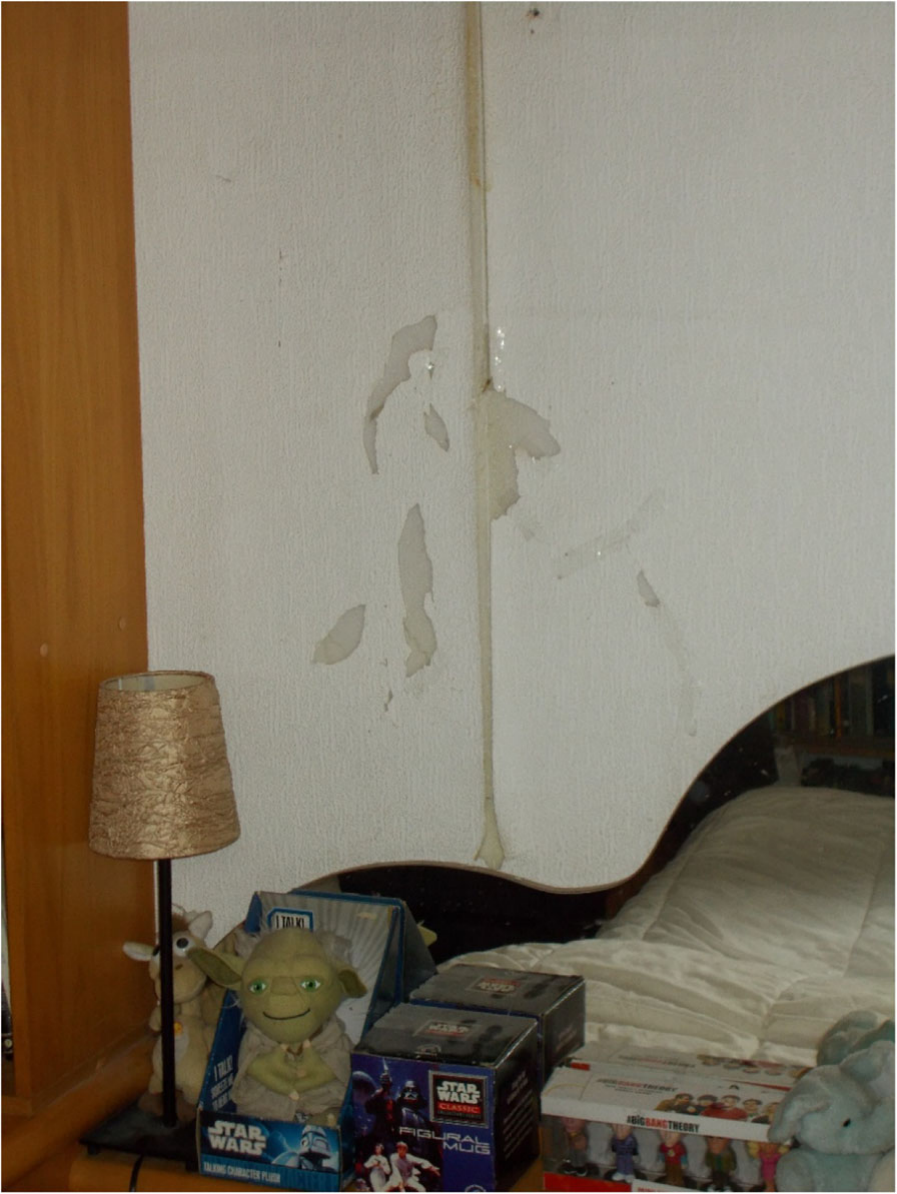
Dampness on the wall (F.H. 57-year-old man)

Participants captured and emphasised that their health was directly affected by dampness, lack of proper insulation (problems with windows and doors), faulty electrical outlets and connections, and inability to deal with maintenance costs due to lack of savings. Physical health is jeopardized due to the risk of electrocution, allergies, and contracting respiratory diseases and infections. Similarly, the distress and shame of seeing one’s house in bad condition can cause psychological harm. M.E., a 61-year-old man, said: “I have 6 o 7 sockets I cannot touch them because if I touch one I will get electrocuted and then I will be in real trouble”, while F.H., a 57-year-old man, explained: “I’m ashamed to invite someone to my house. I have a “partner”, who lives well and sometimes she tells me “let’s see when I go to your house”, and I say “no, you can not come to my house”, and she does not come to my house, because I am really ashamed.” (Fig. 3). According to participants, poor housing conditions resulted from a lack of economic resources and, importantly, because it is not worth investing time and money in a dwelling that you may have to leave at any time.

### Health-related behaviours

Participants observed that unhealthy behaviours have been more common since their housing problems began. The photographs analysed included sedentary behaviour, less self-care (infrequent medical visits and treatment adherence), sleeping problems, unhealthy diet and increased consumption of alcohol and sleeping pills.

**Fig. 4:**
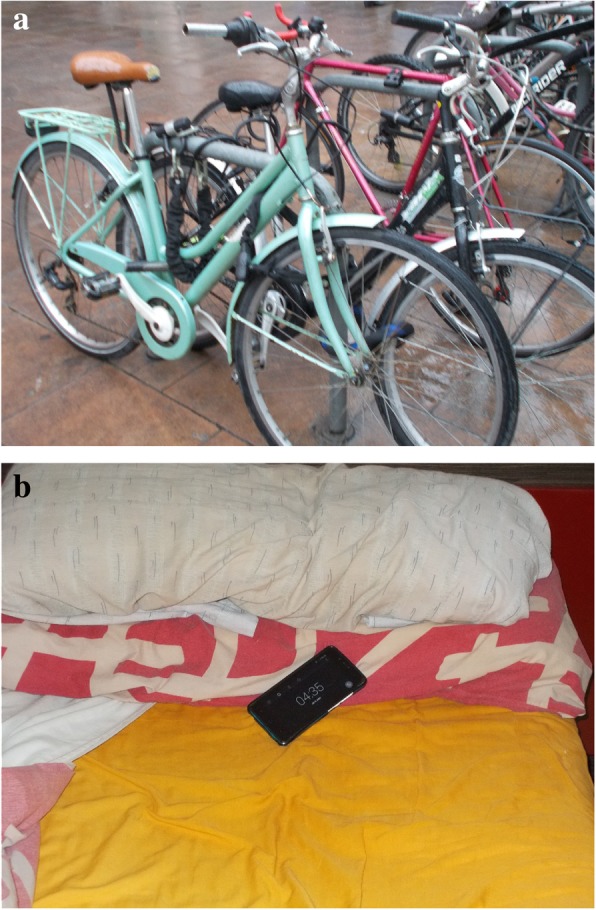
**a** “I miss doing things that I like” (R.N., a 34-year-old woman). **b** Awake at 4 AM! (J.A., 41-year-old man)

Regarding sedentary behavior R.N., a 34-years-old woman, said: “I love sports. I used to bike a lot, but not anymore [since the housing problem]”] (Fig. 4a). In the case of self-care, she added: “I do not go to the doctor, whatever happens to me (...) because the thing about going to the doctor is that I know that the doctor will ask me for lots of things, he will send me to do lots of things. You have to solve this first, because the stability [of the house] is the most serious thing, you know?”

Participants noted that their stressful lives had led them to increase their consumption of alcohol and sleeping pills. Most participants agreed that sleeping problems are very common and affect their normal daily functioning. For instance, J.A., a 41-year-old man, mentioned: “Yes, I have reached that extreme. I take 2 or 3 pills to get to sleep, and it does not take effect. They have already changed [the pills] four or five times, and there is no way, I cannot sleep.” (Fig. 4b).

Unhealthy diet was a widely discussed topic. Participants agreed that they had poorer eating habits since the beginning of their housing insecurity situation, thereby affecting their health. I.G. said: “I have a simple and plain dinner every day of boiled pasta to put something into the stomach, for me and my children. Then one day you get sick, you go to the doctor, they do tests and they say “eh, madam, you have anemia, you lack iron ... Do you know that with your responsabilities, you have to have good nourishment?” But how am I going to be able to put food on the table tomorrow and be able to continue paying [the house]? If they don’t raise my salary but they raise my rent.” Diminishing the quantity and quality of daily meals due to having to choose between paying housing costs and eating well is a very common dilemma among the participants. However, those with children highlight their efforts to maintain an adequate nutrition for them. C.L. explained: “If the children eat, that’s enough. I have to choose and buy for the children. And for myself, we will see. You have to give the children what there is, but these children are already poorly nourished since they were small.”

### Eviction

**Fig. 5:**
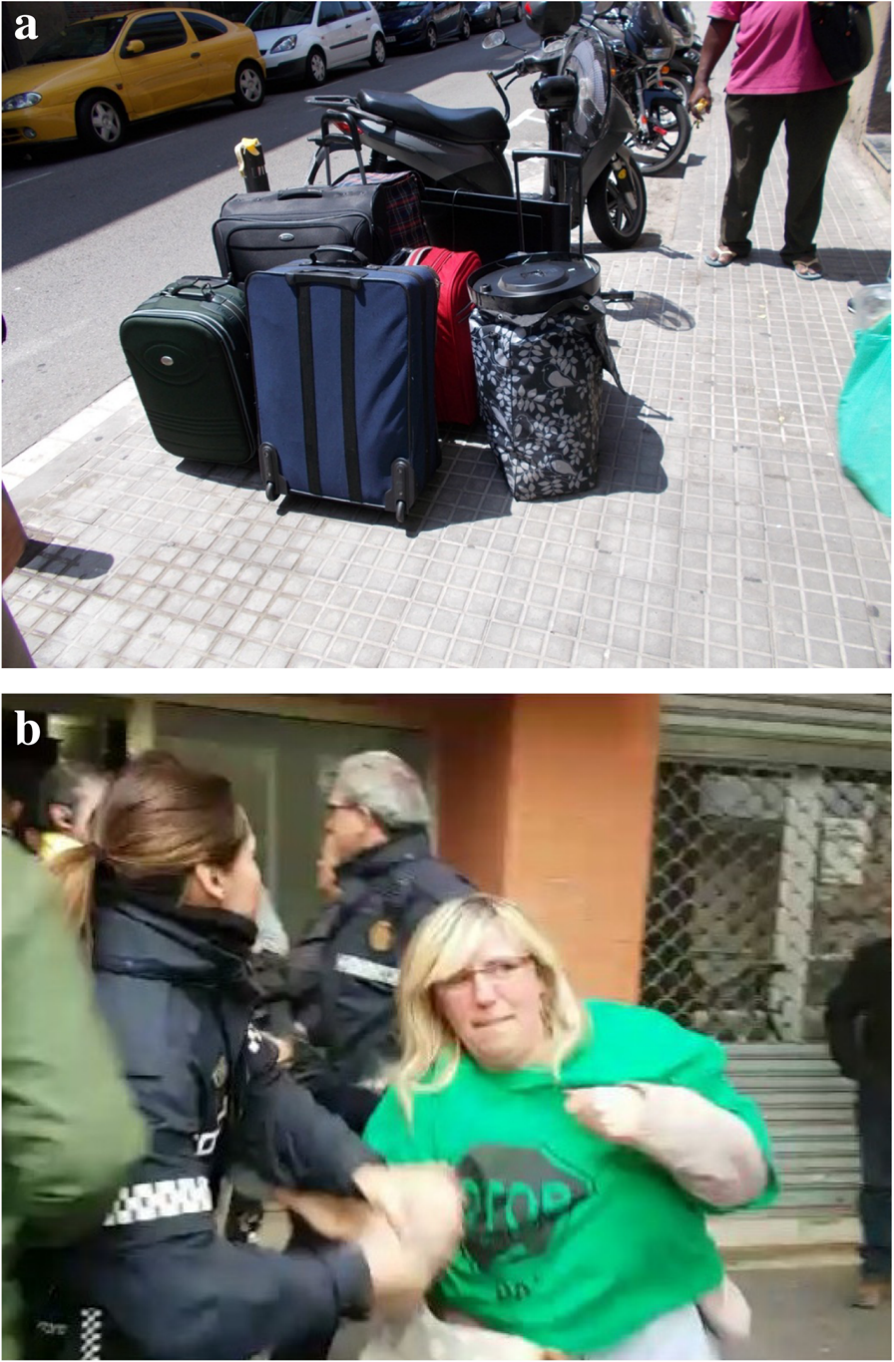
**a** Family stuff on the street (C.L., 40-year-old man). **b** Police intervention (I.G., 49-year-old woman)

Eviction of people from their homes was referred to as a traumatic event that affects both mental and physical health. According to the participants, an eviction triggers anticipatory anxiety and exacerbates stress suffered during the process. Since PAH members participate in preventing evictions of their neighbours, wider groups can also experience the negative effects of evictions, along with those who are directly affected. C.L., a 40-year-old man, said: “Unfortunately, this week we had an eviction that unfortunately could not be stopped. Then, of course, I identified with all this because to see this family, they really leave their things in the street, it reminded me of that for me, because I was afraid of it too, and a week previously I had brought things to a neighbour and went to a room”. (Fig. 5a). In addition, physical health might be affected due to police intervention. I. G., a 49-year-old women, explained: “This [photo] is from this morning, during the eviction of a neighbour. It’s the nervousness that you endure. There, the local police hurt me on the shoulder ... because it was chaotic at that moment when they were being removed, and they violently take you out” (Fig. 5b).

### Harassment by financial institutions

**Fig. 6:**
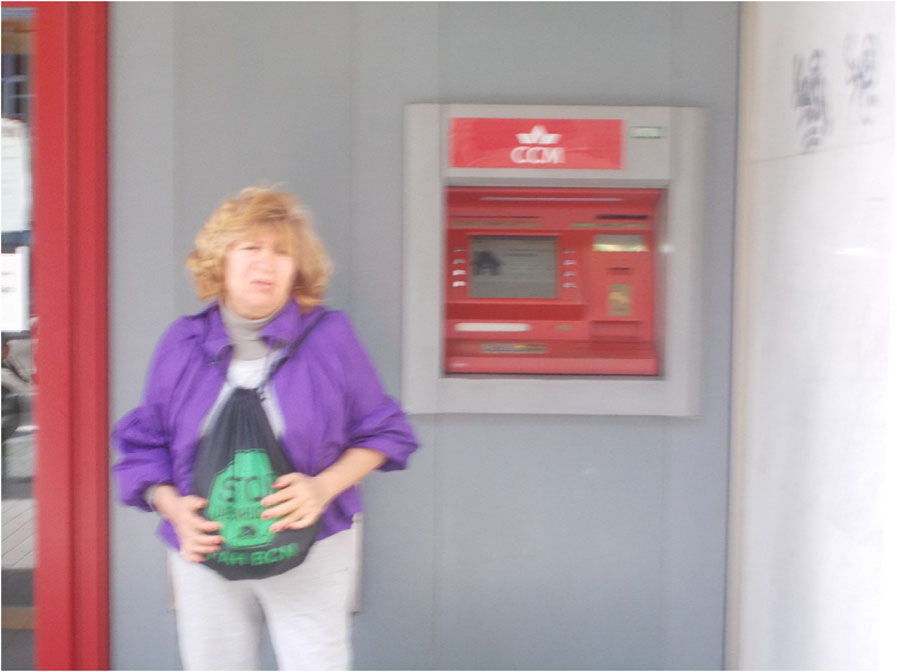
Rage against my bank (L.B., 70-year-old woman)

Regarding financial institutions, participants pointed out that in cases of mortgage and foreclosure, harassment by banks and other non-judicial collection institutions through phone calls, letters and meetings, is a constant burden affecting their mental health and wellbeing. For instance, L.B., a 70-year-old-women, reported anxiety and fear every time she had to meet with bank executives: “I went to the bank to see what solution they could give me and the director first told the lady at the counter that I was leaving, that she was not going to take care of me. She said, “I will not take care of you, after the problems you’ve caused”. That bank causes me anguish because of course, I’m going to fix my problems, and this woman is always so sharp ... and that twists my stomach with anguish” (Fig. 6).

### Family, neighbours and social network

**Fig. 7:**
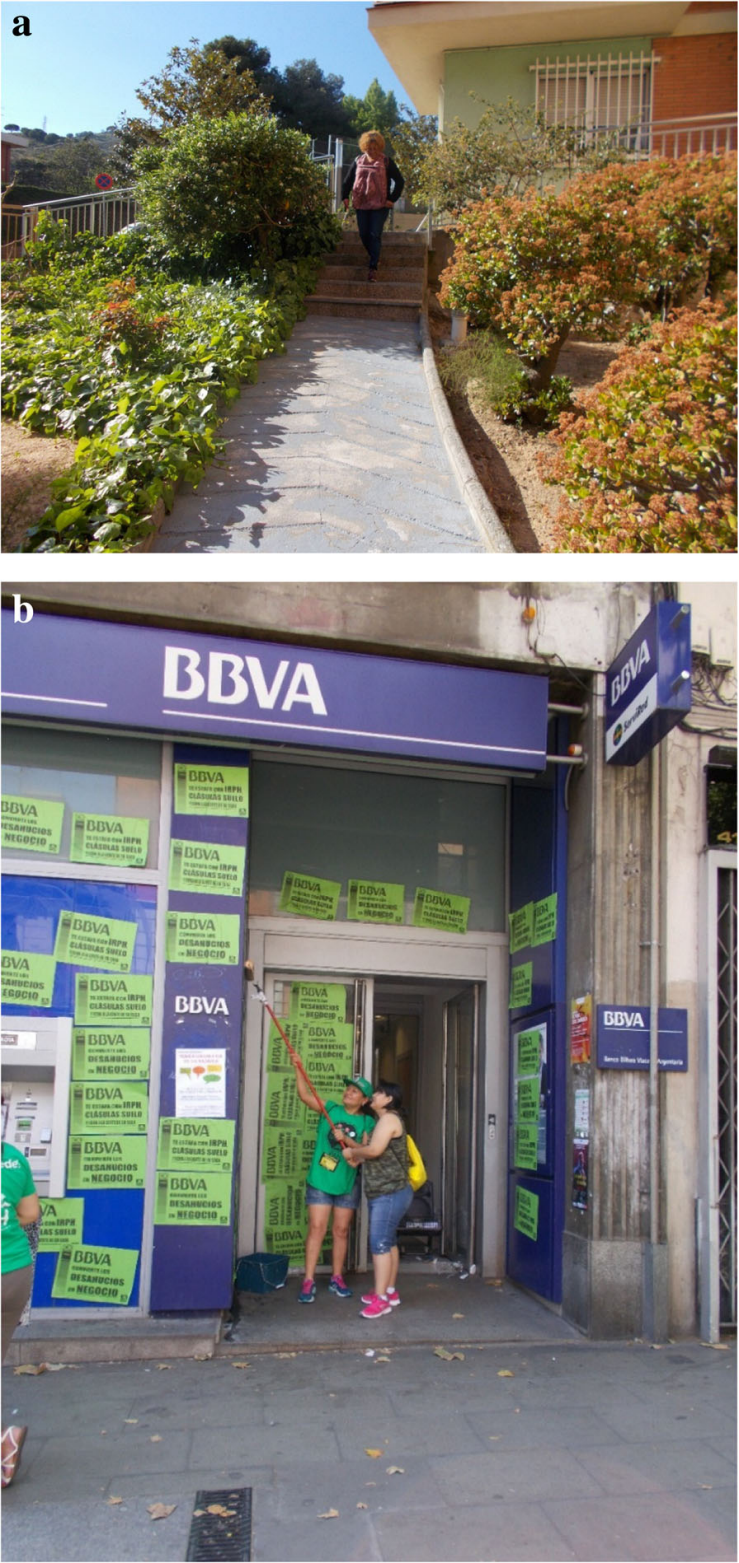
**a** Loneliness (E.V., 55-year-old woman). **b** “Mom, can I help you?” (C.P., 50-year-old woman)

According to participants, their social network is another factor involved in the relationship between housing insecurity and health. Being forced to leave one’s dwelling implies leaving the community and the social network as well. This situation leads to isolation and a lack of social support, which can produce negative effects on mental health, as captured by E.V., a 55 year-old woman, in her photograph (Fig. 7a).

Another aspect is the importance of family. Participants explained that having strong family support lessens the negative effects of the housing crisis on mental health. As C.P. mentioned: “My daughter likes to be there, supporting me, caring for me, because I take her and she joins in. “Mom, I will help you”, we are pasting posters on the walls of the bank (...) it’s a way of ... how can I put it? To seek complicity with me (...) and we show it at home, in actions, in any way we can, we get rid of the discomfort, stress, nerves, powerless, everything” Fig. 7b). However, some participants explained that indifference and rejection by their families and neighbours to their housing problem increased the sense of isolation, shame and guilt. In addition, affected people usually do not share the housing crisis load with their families in order to prevent further suffering (mainly their children), which increases their own stress and sense of isolation.

### Employment and household economy

**Fig. 8:**
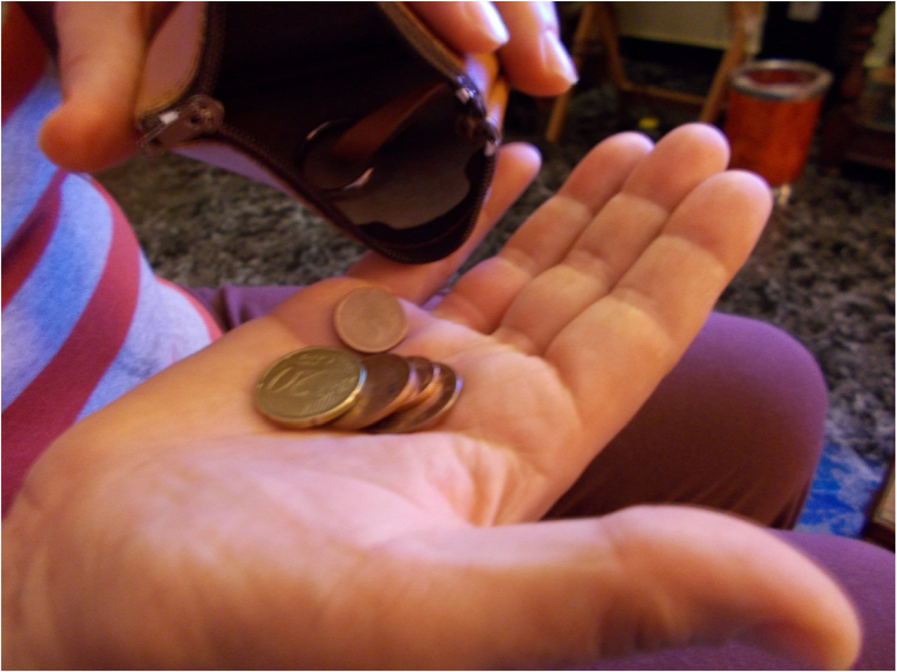
Make ends meet (T.V., 56 year-old-man)

While unemployment and shortage of money are causes, more than mediators, participants agreed that these factors strongly interact with housing insecurity and its effects on health. In all cases, losing a job led to losing the dwelling. Further, the difficulty in finding a new job in a precarious job market makes it harder to have enough resources to overcome housing insecurity, thereby exacerbating the negative consequences on health. As M.L., a 56-year-old woman, added: “I cannot work. I cannot work anymore in my job. So they screwed up my life. Because with my work, I earned money. I had the apartment, I had everything, more or less. But when I could not keep working, what can you do?”. T.V., a 56 year-old man, added: “We cannot make ends meet” (Fig. 8).

The association between unemployment and housing insecurity is often a two-way process. People under threat of eviction said that they spend a lot of their daily time in legal and administrative proceedings such as negotiating with financial institutions, attending social service offices, and meeting with lawyers. I.G. said: “You can go so far as to lose your job to fight for a house, like what happened to me. I lost my shop. I was not doing very well but it was me and it showed, and I have lost my way of life because every day for three months I had go to the housing office, court, social services ... daily, in person”.

### Energy poverty

**Fig. 9:**
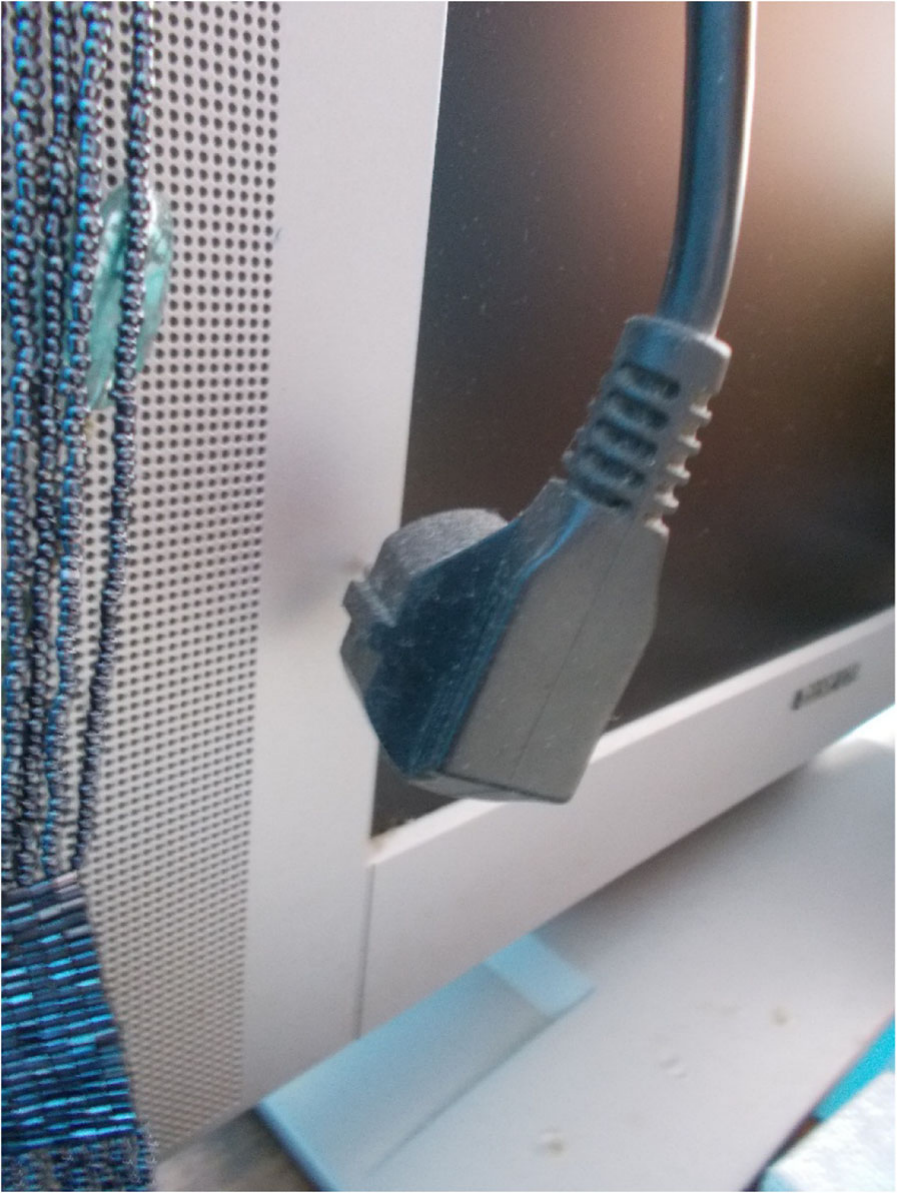
Trapped by the plug (C.G., a 46-year-old woman)

People experiencing housing insecurity often have to choose between paying their rent or mortgage and paying basic amenities (electricity, gas and water). Participants explained that they usually prefer to pay rent before paying for energy, despite saving as much as possible by cutting their energy use prior to making that decision. Therefore a lot of daily provisions, such as heating and food, cannot be afforded, which has negative consequences for their health. As L.B. said: “I do not have air conditioning or heating, so I’m sick of course. It’s cold, and on top of that, I’m sick with bronchitis. This time I’ve been like this for three months in bed, because I’ve had pneumonia, and of course, I can’t, I can’t put on either the heating or the hot water.” In addition, the people affected reported high levels of anxiety due to an imminent cut in their energy supply due to payments arrears. For instance, C.G. mentioned: “I’ve been ‘stuck’ for a long time [using] the plug until I said one day “to hell with it”. I mean, I unplug and that’s it. As I know there is no electricity [standby light], because I unplugged, because I do not have to turn on the TV in the morning to see if they have turned off the electricity or not ... so I’m calmer”. (Fig. 9).

### The PAH

**Fig. 10:**
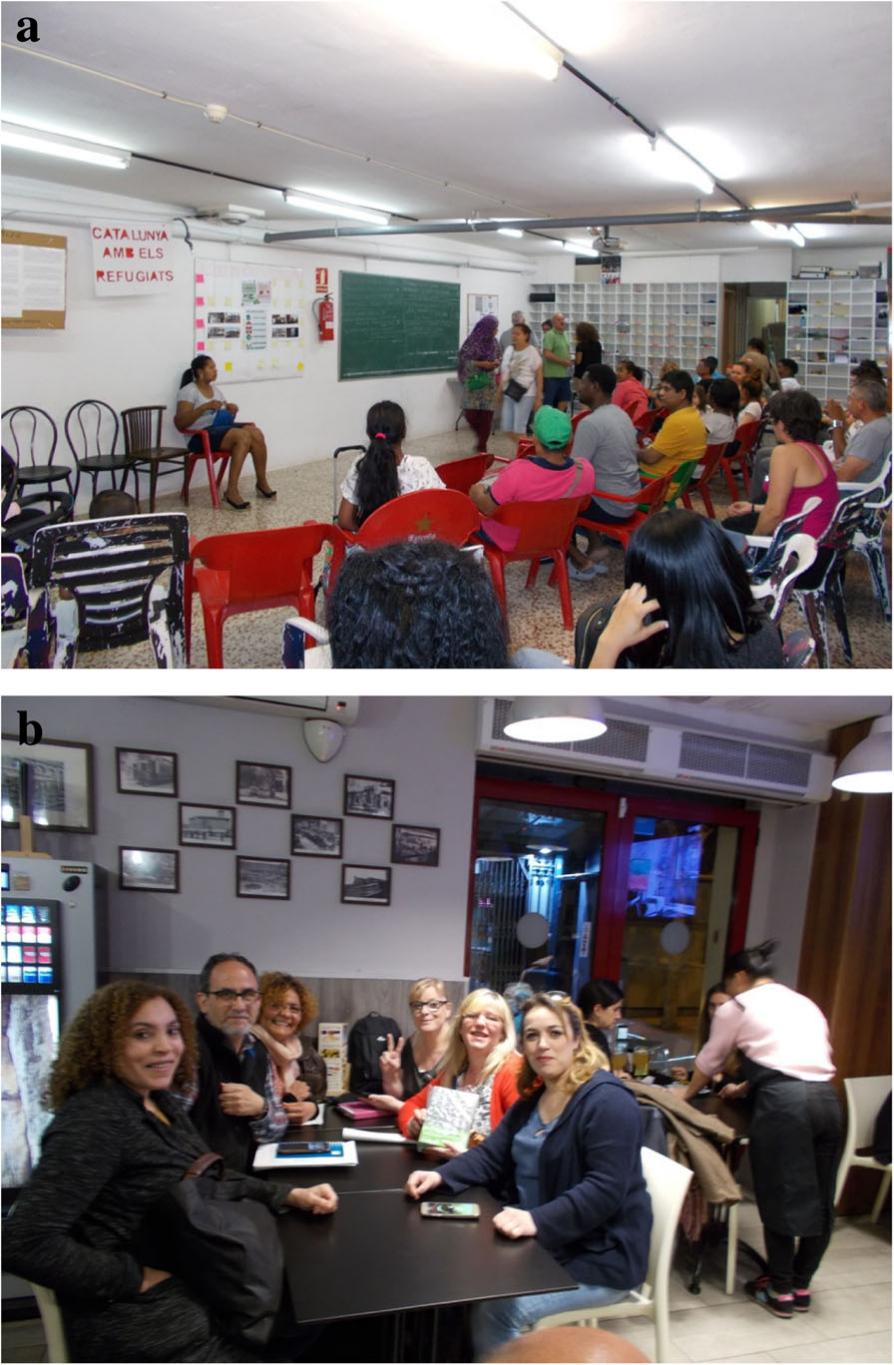
**a** PAH meeting (C.L., 40-year-old man). **b** Hanging out with PAH friends (F.H. 57-year-old man)

All participants agreed that the PAH is the most important protective factor that mitigates the negative effects of housing insecurity on health, by modifying several of the aforementioned mediators, namely preventing eviction, creating new social networks, and empowering people by tackling psychological hardships. Participants reported that people arrive at the PAH emotionally damaged, with feelings of shame, guilt and powerlessness. However once they start to participate, positive changes rapidly occur (Fig. 10a). As T.V. explained: “Here it is clear, and it has been shown to all of us in the PAH, that people arrive broken, the vast majority, absolutely broken, and after a while, because it has quite rapid effects, the truth is, you go out, you move differently. Fortunately”. As participants reported, the PAH gives them tools to become empowered, overcoming shame and guilt and figuring out that they can find solutions for their housing situation together. T.V. added: “We went from a completely depressing state initially, lack of self-esteem, not believing in anything and such, to attending assemblies and meetings, and finding common solutions to fix things a bit.”

Another characteristic of the PAH that diminishes negative health consequences is that collaborative work gives people motivation to take positive action. As L.B. mentioned: “For me it is great therapy because seeing that I can help that person, it is something that enhances me, that is, I am no longer alone, I no longer feel insignificant, I know I can do something positive, something great, for others as well as for me.” The PAH usually provides people with a new social network and a peer support group where they can be understood and helped. This is relevant considering the impact housing insecurity has on the social fabric, as mentioned above. I.G. said: “I have also found friends, maybe not close lifelong friends, but you can have a great friendship coming to the PAH, because here we do not look at race, colour or social status, here we all come to do the same” (Fig. 10b).

### Public services

**Fig. 11:**
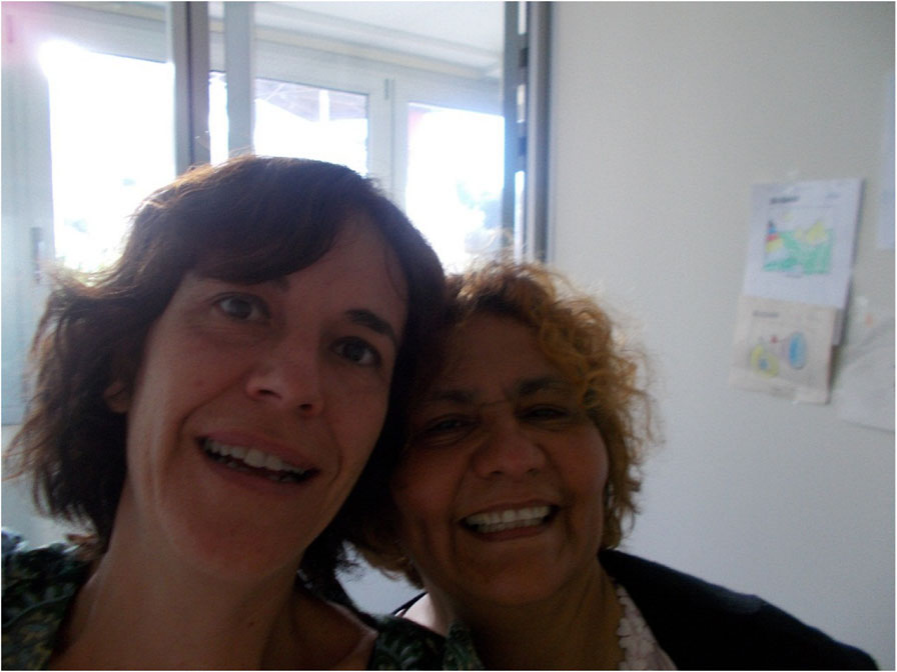
My social worker (E.V., 55-year-old woman)

Both groups agreed that public services, such as primary health care, social services and housing assistance offices might modulate the negative effects on health. Although this topic was considered a point of contention, as the response and attitude of public service personnel can lessen or increase negative health effects among people with housing insecurity. For instance, public servants sometimes make recipients of their services feel guilty about their situation, undermining their self-esteem and mental health. As F.H. explained: “And you have that person there who is dealing with you, you know that she is in a good position and she is treating you as if you do not want to work, like you are practically a squatter, and then you have to explain to her. Lots and lots of these people who are there, they think that we are “living the life”, but we and the people that I know are not like that.” On the other hand, E.V. stated that a warm reception and empathy by public service workers make participants feel supported and understood, which reduces the stress and bad feelings of their situation: “When I met the social worker, it was a service that gave me life ... that woman [the social worker] to me, has given me life, because she has treated me so excellently right up to today, where this case has taken me.” (Fig. 11).

### The current housing market

**Fig. 12:**
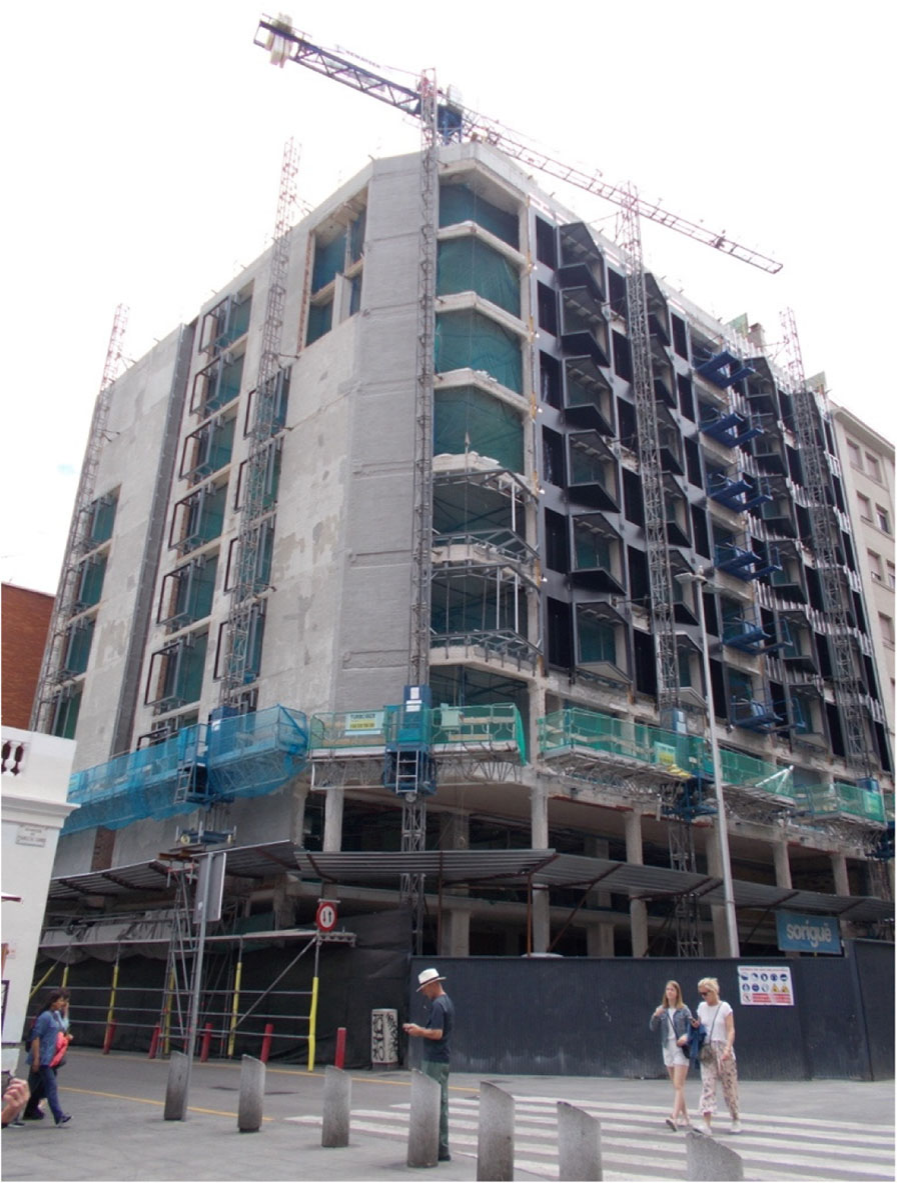
Real estate speculation (C.P., 50-year-old woman)

Although the current situation of the housing market in Barcelona was not recognized as a direct mediator between housing insecurity and health, participants agreed in underlining it as a main structural cause of their residential status. They reported that real-estate speculation, massive tourism and gentrification are leading causes of the housing crisis, and are making them feel angry and powerless. They complained about the increased numbers of dwellings for the investment and tourism markets, instead of being allocated for people who are suffering housing unaffordability and exclusion (Fig. 12). As L.B. said: “This is a cause of the problem, is it not? Because, of course, they are making luxury hotels and flats when there is a lack of housing. I mean, they speculate in making hotels with which they earn a lot, when there are people who do not have a home.”

### Stressful life events

**Fig. 13:**
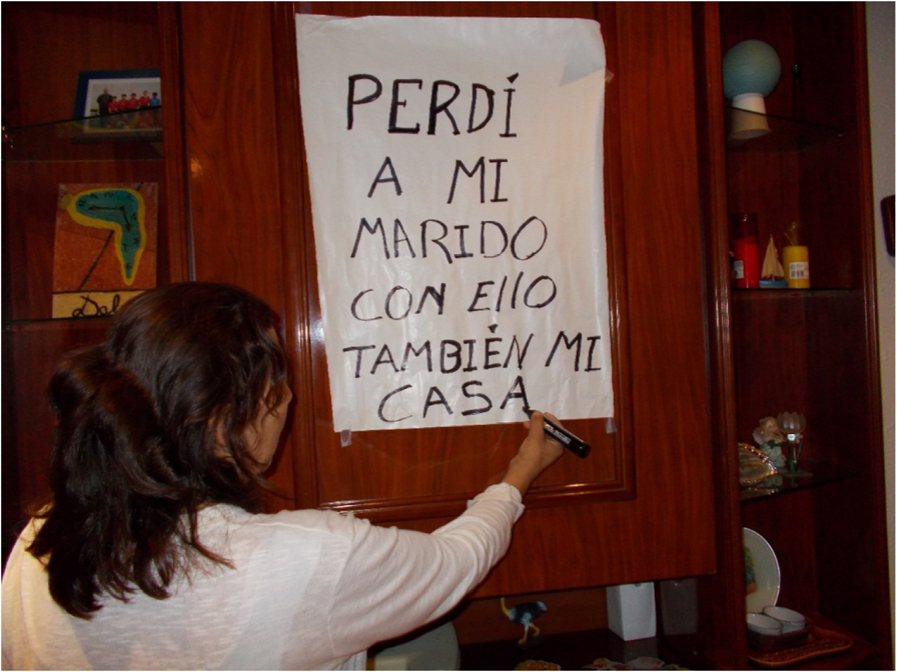
“I lost my husband, with him also my house” (L.C., 55-year-old woman)

Participants reported that stressful life events, such as the death of a relative, family break down or chronic disease are closely linked to negative health consequences. These events can act either as triggers of housing insecurity or as interacting factors that worsen that insecurity. For instance, L.C., a 55-year-old-woman, said: “My husband and I work thinking that we would be in this apartment all our lives, but since my husband died I have a problem. So [in this photograph] I’m imagining that I surely have to leave the apartment”. (Fig. 13).

### Recommendations

The recommendations proposed by the participants to tackle these factors are available in the supplementary data (Additional file 1).

## Discussion

This photovoice project provided insights into the mediators and mechanisms that link housing insecurity and health among people associated with the PAH in Barcelona. Using a participatory approach, participants reported a variety of factors associated with housing insecurity that, in turn, can affect mental and physical health Additionally, participants described how being active in the PAH and the response of public services can modify these factors, with either increased or diminished health effects. Linked to this problem, energy poverty, employment and household economy emerged as social determinants that are tightly related to housing insecurity and could also influence health outcomes. Finally, despite not being directly involved as mediating factors, stressful life events and economic and political causes of housing insecurity emerged as important topics to be considered. All of these results show the unbalance of the participants on social life and function practice of their social role.

Some of the findings on the mediators reported here have been described in previous studies. Regarding psychological changes, some studies have reported that housing insecurity can trigger feelings of personal failure, embarrassment, isolation, uncertainty and having a lack of control over key aspects of daily living which, in turn, can lead to anxiety, depression and suicidal ideation [7, 16, 20–22]. Nettleton and Burrows pointed out that living with housing insecurity affects health via two pathways: 1) through psychological changes, and 2) by adopting unhealthy behaviours that are risk factors for several diseases [16]. The second pathway is also supported by our findings and those of other studies showing that people report more sedentary behaviour, alcohol consumption, sleeping problems and unhealthy diet after becoming affected by housing insecurity [23, 24, 29–31]. The social context, such as housing, influences those individual health-related behaviours in a number of ways: by shaping social norms, enforcing social control, enabling or not enabling people to participate in particular behaviours, reducing or producing stress, and constraining individual choice [32].

Beyond behavioural mediators, this study added other peripheral factors. Regarding family and social network, we found that people with housing insecurity may suffer from lack of empathy, rejection and discrimination from family and neighbours, or forced displacement to other neighbourhoods, which can lead to isolation and loss of their social network. Likewise, Hulse and Saugeres reported that housing insecurity generates barriers to social support and participation (related to high mobility, lack of belonging and isolation), which are widely recognized as determinants of poor health outcomes [7, 33]. Libman et al. reported that the threat of eviction can create enough stress to break up families, while Murphy et al. showed that the association between housing affordability problems or eviction and alcohol dependence symptoms was stronger among people with low perceived family support [29, 34]. Additionally, participants discussed how housing insecurity leads to physical deterioration of the dwelling as a consequence of household economic problems and uncertainty due to an imminent eviction. This finding is supported by studies showing that people affected by housing insecurity are more likely to live in inadequate dwellings, with mould, dampness, and pests, compared to individuals from the most disadvantaged social classes [7, 35]. Most of the literature on mediators between housing insecurity and health has focused on psychosocial pathways. However, our understanding is that several mechanisms are explained by material living conditions that should be addressed by further research. Regarding eviction, participants described it as a highly stressful event that can trigger psychological distress and anxiety, and that is also a potential risk factor for physical health due to police intervention. However, to our knowledge no other studies have addressed this specific issue. The final direct mediator that emerged is harassment by the financial institutions, which increases victims’ feeling of lack of control, fear and anxiety. Although we did not find evidence of this in our study, previous evidence has associated indebtedness to negative effects on health, highlighting that one of the mechanisms involved would be the debt collection process. This process includes formal and informal procedures that generate stress and anxiety among debtors, mainly in cases where creditors have the right to take debtors’ property [36, 37].

Another noteworthy finding was the relationship between energy poverty and employment and housing insecurity. Despite not being direct mediators, participants reported that both determinants are closely linked to housing insecurity and can most likely worsen the negative consequences on health. Regarding energy poverty, Cook et al. reported that young children living in households suffering energy poverty are more likely to suffer housing insecurity and fair or poor health, although they did not analyze possible interactions between both determinants [38]. In turn, Hernández has proposed the term “trifecta of insecurity” to discuss the associations between housing insecurity, energy insecurity and food insecurity, and how this can affect the health of people who are forced to make tradeoffs in meeting basic needs due to socioeconomic constraints [39]. Generally, people living with housing insecurity not only struggle to pay their rent or mortgage, but also high energy bills which may increase the stress or lead to energy poverty [40, 41]. We understand housing insecurity and energy poverty to be pieces of the same phenomenon, and their effects on health and their interactions should be addressed. Participants pointed out that unemployment and other employment insecurity situations are closely associated with housing insecurity and health. Unemployment can directly cause housing insecurity and, conversely, housing insecurity can lead to job loss or can make it difficult to find a new one. For instance, Hulse and Saugeres reported that attempting to find work while experiencing housing insecurity, and not being able to find or keep a job, reinforced the stress, anxiety and depression that respondents experienced [7]. Bentley et al. studied these interactions in a sample of people of working age, and reported that employment security modifies the relationship between housing affordability and mental health. The negative effects of unaffordable housing on health were more intense among people under insecure employment conditions [42].

The response of public services and participation in community-based organizations, such as the PAH, emerged as two important modifiers of the effects of housing insecurity on health. They help alleviate some of the described mediators and pathways of poor health. Social services can improve household income through subsidies, promote social inclusion of affected families, provide legal and administrative solutions to stop evictions and integrate affected people into the social protection system. Primary health care centres may address health-related behaviours and psychological changes using a comprehensive approach. Housing offices can offer social housing, rent subsidies and other measures to assist people under housing insecurity. In contrast, lack of empathy and inadequate responses from public services can worsen the feelings associated with housing insecurity among the recipients, leading to poorer mental health outcomes. In addition, participants agreed that the lack of specific training in housing insecurity phenomenon for public services personnel and problems related to the efficiency of bureaucratic processes (mainly in Social Services and Housing offices) jeopardize a timely response to urgent issues related to material living conditions (e.g. food insecurity, energy poverty, lack of shelter) which can be another mechanism to poor health. There was unanimous agreement among participants that the PAH was the greatest protective factor. Peer-to-peer support, empowerment and social inclusion were described as the first steps in easing feelings of guilt, embarrassment and personal failure. The PAH carries out several actions to tackle housing insecurity. Evictions can be prevented through nonviolent resistance. Collective repayment negotiations are carried out with banks. Evicted tenants are re-housed, ideally in nearby neighbourhoods. Attempts are made to push for revision of local and national laws to change the unfair conditions of the housing system. In combination, all of these actions modulate most of the described mediating factors, reducing the negatives effects on health [43]. In addition, PAH promotes the political empowerment of its members by disrupting the hegemonic neoliberal model since people realize that losing their houses is not their personal failure but is linked to structural factors [17]. As seen in this and previous studies, social and political participation is crucial to improve inadequate and unfair living conditions, and to improve the health of the affected population [33, 44].

This study used a Participatory Action Research approach, in which a Photovoice methodology engaged participants and other PAH members who themselves made decisions and validated results from the beginning. This allowed us to obtain reliable information about the pathways to poor health of a hard-to-reach section of the general population. In addition, the study has produced political recommendations for interventions by the PAH, such as advocacy actions with policymakers and other stakeholders (Additional file 1). Regarding the study’s limitations, the recruitment process did not allow us to create gender-stratified groups, which limited our ability to gather gender-specific views. The definition of “home” is inherently gendered, and so further research that takes gender differences into account is necessary. These findings are not representative of all people experiencing housing insecurity in Barcelona, as they were obtained from a specific sample, namely those associated with the PAH. For instance, probably if participants were not PAH members (or another social movement) the importance of collectivism (i.e. the PAH) and the political empowerment highlighted on some categories (e.g. the current housing market), would not be that much relevant as in this case. However, considering the difficulty in accessing people living with housing insecurity, we consider this study an appropriate initial approach to identify and further characterize the problem and its links to health. Finally, while we present the main findings in Fig. 1, the lack of a conceptual framework on housing insecurity and health has hindered understanding of the complex associations we have identified. We highlight the need for further research and theory in this field.

## Conclusion

Housing insecurity in Spain, and many other places, is far from being resolved and will likely continue in the coming years. This situation clearly affects the population’s health and exacerbates social and health inequalities. Understanding the pathways and mechanisms underlying this process is a crucial task of social epidemiology. Further research in this field and public engagement are urgently needed to design and implement public policies that tackle this serious problem.

## Additional file

Additional file 1: Recommendations for tackling negative effects of housing insecurity on health. (DOCX 20 kb)
