# Supplementary material for: Our lives in boxes: perceived community mediators between housing insecurity and health using a PHOTOVOICE approach
Source: Int J Equity Health. 2019 Mar 27;18:52. doi: 10.1186/s12939-019-0943-0 (PMC6438010; doi:10.1186/s12939-019-0943-0)
Supplement: Supplementary file 1 — Recommendations for tackling negative effects of housing insecurity on health. (DOCX 20 kb) [file 12939_2019_943_MOESM1_ESM.docx]

**Additional file 1:**

**Recommendations for tackling negative effects of housing insecurity on health**

The recommendations that emerged from the participatory process are listed below according to each of the identified categories:

**Mediators between housing insecurity and health**

Psychological changes

1. Stepped psychological care in health centres that promotes mutual support groups and collective therapy:
   1. Collective work is considered necessary, creating spaces of emotional support (i.e. peer-to-peer support).
   2. Priority individualized psychological care (without delays) for severe cases.
2. Decrease pharmacological prescription for emotional distress and instead promote alternative techniques such as physical activity, relaxation techniques and yoga.

Housing related material aspects

1. Lengthen the duration of rental contracts. This would allow tenants to feel more rooted and secure and so encourage them to invest money and time in dwelling repairs.
2. Create dwelling refurbishing subsidies for people affected by housing insecurity.

The eviction

1. Prohibit evictions, establishing different actions according to:
   1. Banks, financial institutions and big landlords: In this case, evicting families should be prohibited. Creditors/landlords should offer solutions such as relocating the affected family or renegotiating debt payments.
   2. Small landlords: In this case the eviction should be prohibited until there is a relocation alternative for the affected family. It implies an efficient intervention by the Housing and Social Services offices from the beginning of the process.

Family, neighbours and social network

1. Relocate evicted families in the same neighbourhood, or as close as possible, to avoid breaking their social network.
2. Include housing insecurity as a theme into schools’ formative programs to make this problem visible in the community and to be able to work with affected children and their families.
3. Create awareness campaigns in the neighbourhoods so that people not affected by the housing crisis understand and empathize with the situation of their affected neighbours.

Health-related behaviours

1. Sensitize and train primary health care personnel on housing insecurity and how social issues affect health and health-related behaviours to avoid “victim blaming”.
2. Promote social prescribing in health care centres (e.g. referral to non-clinical community resources such as dance workshops, volunteering, artistic activities, urban gardens).
3. Ensure that people affected by housing insecurity have access to free cultural, leisure and sports spaces in their neighbourhoods.
4. Promote coordination between health care centres and Social Services, Housing offices, and other institutions involved.

Financial institutions

1. In the face of harassment and mistreatment by financial institutions, the competent institutions (Síndic de Greuges and the Catalan Consumers Office) should ensure that people are treated with respect and dignity.
2. Administration should promote education campaigns on consumer rights so that affected people have more tools to deal with banks.
3. Harassment (by phone and in person) by financial institutions should be prohibited by law.

**Modifiers of the mediators’ effects**

Public services

1. Improve the efficiency of bureaucratic processes in public service offices (mainly in Social Services and Housing offices).
2. Specific training in the housing insecurity phenomenon for public services personnel who attend affected people to improve treatment and reception.
3. There should be adequate turnover of public services front-line staff to avoid emotional burnout and, in turn, possible mistreatment of affected people who come for help.
4. Ensure the implementation of more humanized care protocols. Those protocols should ensure that people receive information about all social rights they have access to, to avoid the feelings of paternalism or being receiving charity in their first visit.
5. Create collective attention systems in public services.

The PAH

1. Include the PAH in the various bodies that may influence political decisions, such as consultative or technical councils of the Administration.
2. Increase the number of communication campaigns to publicize the work of the PAH on housing rights.

**Co-existing determinants involved in the relationship between housing insecurity and health**

Energy poverty

1. Demand compliance with Law 24/2015 (measures to tackle housing insecurity and energy poverty in Catalonia).
2. Cancel debts on basic utilities (i.e. electricity, water and gas) of people affected by housing insecurity and energy poverty.
3. Guarantee basic utilities (i.e. electricity, water and gas), especially for families affected by housing insecurity.

Employment and household economy

1. Ensure a guaranteed minimum income to allows a dignified life.
2. Ensure quality employment.
3. Facilitate the employability of less qualified people.
